# Supplementary figures and images for: Lipopolysaccharide Administration Alters Extracellular Vesicles in Cell Lines and Mice
Source: Curr Microbiol. 2021 Feb 9;78(3):920–31. doi: 10.1007/s00284-021-02348-5 (PMC7952295; doi:10.1007/s00284-021-02348-5)

## Slide 1
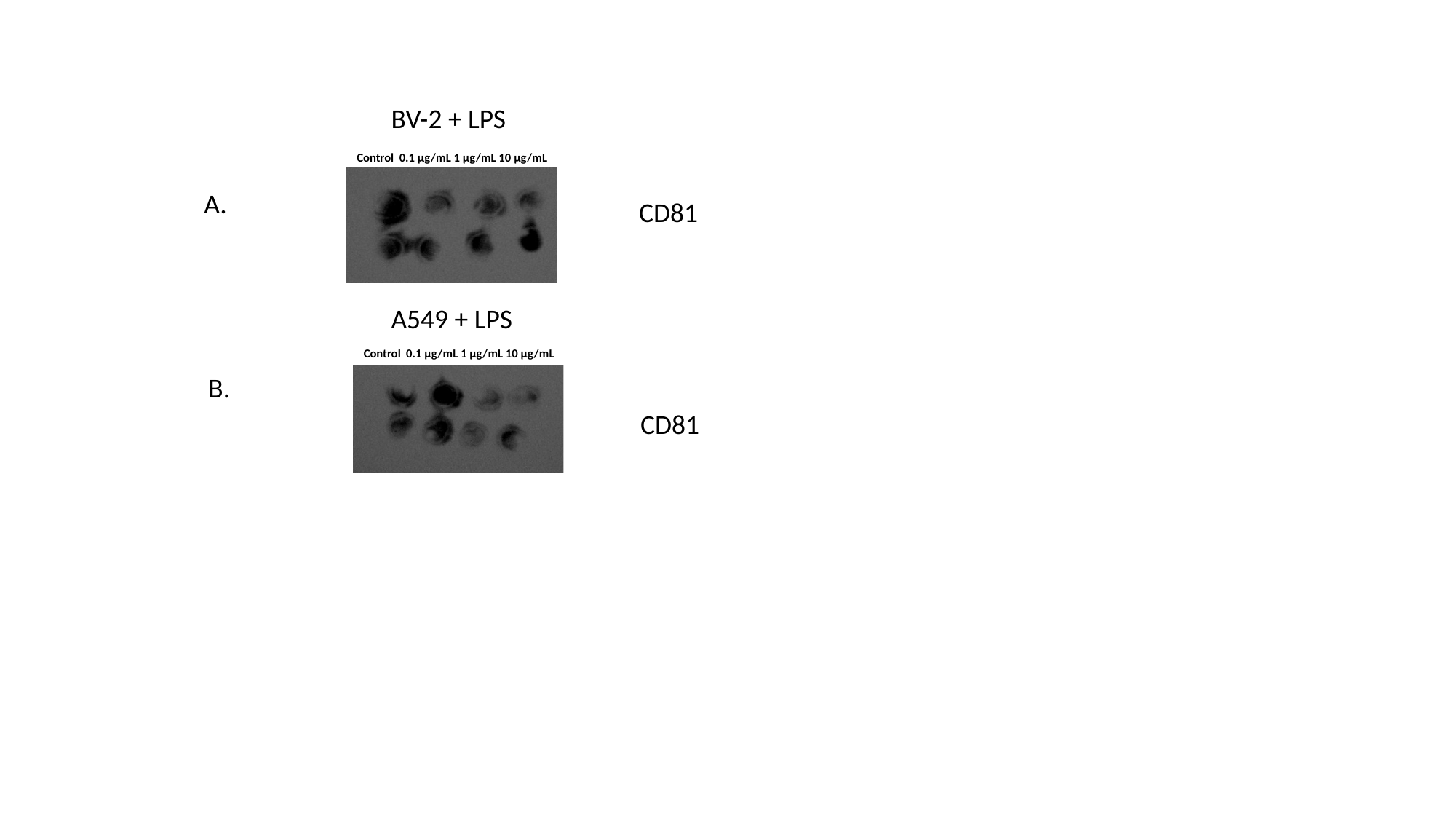

BV-2 + LPS
Control 0.1 µg/mL 1 µg/mL 10 µg/mL
A.
CD81
A549 + LPS
Control 0.1 µg/mL 1 µg/mL 10 µg/mL
B.
CD81

Supplement: Supplementary file 1 — Electronic supplementary material 1 Supplemental Figure 1. Expression of an EV-associated protein in BV-2 and A549 cell lysates. Dot blot analyses of CD81 in BV-2 and A549 cell lysates following LPS treatment (0.1 µg/mL, 1 µg/mL, and 10 µg/mL). (PPTX 617 kb) [file 284_2021_2348_MOESM1_ESM.pptx]
